# Involuntary weight loss in elderly individuals: assessment and treatment

**DOI:** 10.1590/S1516-31802001000200007

**Published:** 2001-03-02

**Authors:** Julio Cesar Moriguti, Eny Kiyomi Uemura Moriguti, Eduardo Ferriolli, João de Castilho Cação, Nelson Iucif, Julio Sergio Marchini

**Keywords:** Elderly, Weight loss, Disability, Mortality, Idoso, Perda de peso, Incapacidade, mortalidade

## Abstract

**CONTEXT:**

The loss of body weight and fat late in life is associated with premature death and increased risk of disability, even after excluding elderly subjects who have a preexisting disease. Although it is important to recognize that periods of substantially positive or negative energy balance and body weight fluctuation occur as a normal part of life, weight losses greater than 5% over 6 months should be investigated. We can divide the major causes of weight loss in the elderly into 4 categories: social, psychiatric, due to medical conditions, and age-related. The clinical evaluation should include a careful history and physical examination. If these fail to provide clues to the weight loss, simple diagnostic tests are indicated. A period of watchful waiting is preferable to blind pursuit of additional diagnostic testing that may yield few useful data, if the results of these initial tests are normal. The first step in managing patients with weight loss is to identify and treat any specific causative or contributing conditions and to provide nutritional support when indicated. Non-orexigenic drugs have found an established place in the management of protein-energy malnutrition. Early attention to nutrition and prevention of weight loss during periods of acute stress, particularly during hospitalization, may be extremely important, as efforts directed at re-feeding are often unsuccessful.

**DESIGN:**

Narrative review.

## RESUMO

**CONTEXTO:**

Perda de peso e de gordura na velhice está associada com morte prematura, aumento do risco de incapacidade, inclusive após exclusão daqueles idosos que têm doença preexistente. Embora seja importante reconhecer que períodos de flutuação substancial do balanço energético positivo ou negativo e peso corporal ocorrem como parte normal da vida, uma perda de peso equivalente a 5% ou mais do peso corporal, em um período de 6, deve ser investigada. Podemos dividir as maiores causas de perda de peso em 4 categorias: social, psiquiátrica, clínica e relacionada à idade. A avaliação clínica deve incluir uma cuidadosa história clínica e exame físico. Caso eles não sejam suficientes para o diagnóstico, alguns testes laboratoriais são indicados. Se os resultados dos testes laboratoriais são normais, é preferível um período de observação à uma investigação sem critérios que poderia ser pouco útil. O primeiro passo no manejo dos pacientes com perda de peso é identificar e tratar uma causa específica ou fatores associados e promover um suporte nutricional, se indicado. Nenhuma droga orexígena tem indicação incontestável na conduta de um paciente com desnutrição protéico-calórica. Uma atenção precoce à nutrição e prevenção da perda de peso durante os períodos de trauma agudo, particularmente durante internações hospitalares, pode ser extremamente útil já que os esforços despendidos para uma realimentação, freqüentemente apresentam resultados frustrantes.

**TIPO DE ESTUDO:**

Relato de caso.

## INTRODUCTION

Over the last century, average life span in Brazil has increased from 33.7 to 63.5 years, and the proportion of the population older than 65 years has also increased significantly. It is projected that this population will continue to increase from 7% of total population in 1980 to 16% by 2025.^1^ Therefore, this fact will have an important impact on public health for Brazilians, in that aging has a relationship with increased hospital admission, among other things.^2^

Health maintenance and aging without disabilities are goals for everybody. Aging is associated with progressive changes in body composition that have an important impact on health. Through middle age, there is typically a doubling in body fat content that is associated with a substantial increase in the prevalence of obesity, premature death and disability.^3^ In contrast, after the age of 65 to 70 years, body fat content tends to decrease, even in healthy individuals,^4,5^ and unexplained weight loss leading to protein-energy malnutrition becomes increasingly common.^6,7^ This loss of body weight and fat late in life is associated with premature death, micronutrient deficiencies, frailty, increased hospital admission and an increased risk of disability from falls, and it is also known to delay recovery from injury.^8-11^ Elderly individuals who experience weight loss are at increased risk of premature death and disability, even after excluding those who have a preexisting disease.^9-11^

This article reviews basic considerations regarding the definition, etiologies and clinical significance of weight loss in elderly populations and considerations in their treatment.

## DEFINITION

It is important to recognize that periods of substantially positive or negative energy balance and body weight fluctuation occur as a normal part of life.^12^ However, a weight loss greater than the normal fluctuation should be investigated. Lean body mass declines at a rate of 0.3 kg/year, beginning in the third decade. This decrease in lean body mass tends to be offset by an increase in body fat, which continues until at least age 65 to 70.^13,14^ The end results of these changes are that total body weight tends to peak in the fifth to sixth decade, remaining stable until age 65 to 70. After the seventh decade the elderly subject tends to develop very small decrements in weight at a rate of 0.1-0.2 kg/year.^15^ Therefore, weight loss should not be dismissed as part of the aging process.

Although no clear consensus exists, the best accepted definition for clinically important weight loss is about 5% over 6 to 12 months.^16,17^ Therefore, all weight loss of 5% over 6 months should be investigated.

Even though it may be helpful to inquire if weight loss was volitional, researchers have suggested that all weight loss, whether voluntary or involuntary, is similarly associated with increased mortality.^18^ Among dieters, given the difficulty of maintaining weight loss, clinicians must consider that the ones who are successfully losing weight and keeping it off easily, could be doing so as a result of underlying illness,^19^ although there are numerous possible explanations for this observation.

## ETIOLOGY

The causes of unintentional weight loss have been categorized in several ways. The most basic physiological approach divides the causes into three categories: decreased food intake, accelerated metabolism, and increased loss of calories. In a more descriptive breakdown, Wise^20^ categorizes the major causes of weight loss in the elderly as social, psychiatric, medical, and age-related (Table 1).

### Social

Poverty, living alone and emotional isolation may result in inadequate food intake. Not only may they lack motivation to prepare a meal that only they will eat, but they may have to choose between spending money on food or on prescription medications. A frequent problem among the elderly is the emotional isolation due to the loss of a partner or close friends. Although other family members, such as children, grandchildren, and other relatives, may be present or nearby, they do not fill the gap left by the departure of one or more significant other. This emotional isolation is detrimental to health, and mortality has been well demonstrated. In addition, other elderly people, whether living alone or with a spouse or other relatives, may be at nutritional risk due to lack of knowledge about appropriate foods and food preparation. Many elderly males have neither done the grocery shopping nor learned how to prepare a meal. When a wife or other female relative is not available, they lack the requisite skills to plan and prepare nutritious meals.^21,22^

### Psychiatric

Weight loss arising from dementia is the result of a complex process involving the early olfactory impairment that occurs in this disorder, as well as centrally mediated deficits in the control of appetite and satiety. Some patients with dementia, particularly at moderate stages of Alzheimer's Disease, can lose weight although they increase food intake (they forget that they just ate and then they eat again). This fact could be partially justified by an increase in physical activity (agitation). As the disease progresses, self-feeding skills are lost and dysphagia develops. Major depression may be accompanied by weight change, but weight loss is more common in the elderly population and is accompanied often by anorexia.^20,23,24^ Bereavement is associated with an alteration in the social environment and a decrease in the importance of food, leading to a decrease in energy intake.^25^ Anorexia nervosa can occur in older persons who were previously weight restrictors.^26^ Dally^27^ suggested that "anorexia tardive" may be a more appropriate term for this condition. Several other psychiatric conditions have been associated with weight loss in older people.^28^ These include alcoholism, manipulation (lack of food ingestion used as manipulative behavior), cholesterol phobia, and choking phobia. Many older persons reduce their food intake a few months before death. In some of these patients, life appears to have become an excessive burden and lack of food intake an ethically acceptable method to exit life.^29^

### Medical

Many medical conditions can cause weight loss. Congestive heart failure induces weight loss through its associated lack of gastrointestinal tract motility, hepatic congestion, increased work of breathing, fatigue and weakness due to poorly perfused muscle tissue, and mild protein-losing enteropathy. ^20^ McMurray^30^ et al. found increased concentrations of tumor necrosis factor (cachetin) in a significant proportion of patients with chronic congestive heart failure; the investigators concluded that the factor may, in part, be responsible for cardiac cachexia. Chronic obstructive pulmonary disease has been associated with increased resting metabolic rates due to the increased activity of the respiratory muscle.^29^ Parkinson's disease is associated with anorexia and increased energy expenditure.^31^ Malignant disease can produce weight loss through a variety of mechanisms. Gallstones can produce weight loss secondary to early satiation.^29^ Hypercalcemia is a reversible cause of pure anorexia.^32^ Hyperthyroidism and pleo-chromocytosis are associated with increased metabolism.^29^

Dysphagia is a common problem in older adults, most often due to neurological (Parkinson's disease, Alzheimer's disease, stroke) or esophageal disorders (motility problems, esophagitis, tumors). Dysgeusia refers to impaired taste, and it has been suggested that age-related chemosensory losses play a substantial role in the anorexia that is often observed in older people.^33^ Flavor perception and food recognition are often impaired in older individuals, probably due more to olfactory rather than gustatory sensory loss.^34^ Although olfactory deficits have been shown to correlate with nutritional parameters in healthy older adults, it is not clear that age-related loss of these hedonic qualities alone is sufficient to cause weight loss.^35^ However, medical problems and their treatments can have more dramatic affects on taste and smell, and may increase the risk of malnutrition.^34^ Reversible causes of dysgeusia that should be considered include medications (antihistamines, captopril, carbamazepine, allopurinol, levodopa, clofibrate, lithium, baclofen, and others) and less commonly, zinc deficiency.^36^ Malabsorption produced by bacterial overgrowth, gluten enteropathy or pancreatic insufficiency is also associated with weight loss.^29^

The side effects of drugs are a major cause of weight loss in older people.^29^ Certain drugs cause weight loss by decreasing appetite (digoxin); by causing malabsorption (sorbitol in theophylline elixir); by increasing metabolism (excess thyroxine replacement); or by a combination of anorexia and increased metabolism (theophylline). Therapeutic diets have also been associated with development of proteinenergy malnutrition in older people.^29^

Oral cavity problems have been associated with decreased caloric intake and nutritional deficits.^34,37-39^ Common oral health problems in older adults include dry mouth (xerostomia, often due to medications with anticolinergic effects, or less frequently, Sjögren's syndrome), poor dentition, periodontal disease, and denture and temporomandibular joint problems, all of which can adversely affect chewing and eating processes.^34,38^ Whether these factors alone can cause significant weight loss is unclear, but as common and remediable conditions that have been shown to influence food intake, they merit evaluation and treatment.

**Table 1:** Major causes of involuntary weight loss in the elderly

**Social**
Poverty
Living alone
Emotional isolation
Poor nutritional knowledge
**Psychiatric**
Dementia
Depression
Bereavement
Anorexia nervosa or tardive
Alcoholism
Manipulation
Cholesterol phobia
Choking phobia
**Medical**
Major organ-system disease
Pharmacological effects
Problems of dentition, swallowing,
and chewing
Functional disability
**Age-related**
Impaired olfactory and taste sensitivity
Appetite suppression (central nervous system)

*Adapted from Wise.^20^*

Functional disability (physical, cognitive, and psychosocial) is among the most common contributors to undernutrition in older adults, and it may also be the most frequently overlooked by physicians. The evaluation of weight loss in the elderly must include assessment of economic resources and degree of social sup-port.^17,34,40,41^ The presence of disability or functional dependency is also highly correlated with dietary adequacy, suggesting the importance of physical function assessment and provision of assistance as needed.^40,42^

### Age-related

In general, the elderly present a decrease in food intake accompanied by early satiation.

The early satiation appears to be predominantly due to a decrease in adaptive relaxation of the fundus of the stomach resulting in early antral filling.^43^ Increased levels and effectiveness of cholecystokinin also play a role in the anorexia of aging. Leptin levels (decreasing food intake and increasing metabolic rate) increase with aging in males. With regard to the central feeding drive, both the opiate and neuropeptide Y effects appear to decline with age.^44^

Robbins^45^ has popularized a mnemonic consisting of nine Ds to describe the common causes of weight loss in geriatric population (Table 2).

**Table 2:** The 9 Ds of weight loss in the elderly

Dementia
Depression
Disease (acute and chronic)
Dysphagia
Dysgeusia
Diarrhea
Drugs
Dentition
Dysfunction (functional disability)

*After Robbins.^45^*

## CLINICAL EVALUATION

Because underdiagnosis of poor nutrition and weight loss appears to be a problem, some have recommended nutritional screening for all older adults^17,46,47^ Although numerous screening tests have been developed to detect risk factors for, and presence of poor nutrition, it appears that such tools may not allow for earlier and more effective intervention than can be achieved by close monitoring of body weight.^48^ However, as a minimum, a consideration of medical, psychological, and functional factors that can affect nutritional status should be part of any comprehensive geriatric evaluation.

For those older adults who have lost weight, identified directly (by measuring body weight) or indirectly (e.g. the clothes became too big) their weight loss should first be confirmed. Follow-up weight checks should then be undertaken at weekly intervals.^49^ The first step in the work-up of weight loss is to obtain a history from the elderly person, when possible, and family members. A careful history and physical examination leading to appropriate laboratory testing will identify the causes of weight loss in the majority of subjects.^50^ In taking the history it is important to determine whether the following are present: weakness, change in the ability to taste, olfactory changes, abdominal pain, decreased appetite, nausea, vomiting, diarrhea, constipation, dysphagia, problems with dentition, tobacco and alcohol use, and changes in mental or functional status.^49^ A detailed medication history is also necessary. On physical examination, one may find obvious signs of protein energy undernutrition (alopecia, edema, glossitis, skin desquamation),^49^ and lymphadenopathy.^20^ Neurological examination with mental status assessment (Geriatric Depression Scale and Mini Mental State Examination)^51,52^ completes the

### initial evaluation.

The elderly person with weight loss should be observed while eating. This part of the evaluation should be quite detailed and include where and how the patient is positioned at the table; if self-fed, any difficulties with managing silverware and cups; time spent eating; how much of the food offered is eaten; any chewing problems; any difficulties swallowing; any visual difficulties that interfere with feeding oneself; and qualities of food offered that make it appetizing and appealing to the patient.^49^

During the history and physical examination, special attention should be given to an investigation of cancer, because the prevalence of this disease increases with aging, especially in the lungs, gastrointestinal tract and genitourinary system.

If direct history taking and physical examination fail to provide clues to the cause of weight loss, simple diagnostic testing is indicated (Table 3).

**Table 3:** Initial diagnostic work-up for involuntary weight loss in elderly subjects

**All Patients**
Complete blood cell count
Erythrocyte sedimentation rate
Urinalysis
Renal function tests
Levels of liver enzymes
Albumin
Calcium and phosphorus
Electrolytes
Blood sugar
Thyroid hormones
Chest film
Tuberculosis test
HIV testing, if risk factors are present
**In the absence of localized symptoms, these are indicated**
Fecal occult blood testing
Flexible sigmoidoscopy
Cervical Papanicolaou smear
Mammography
Prostate-specific antigen

*Adapted from Wise.^20^*

If the results of these initial tests are normal, a period of watchful waiting is preferable to blind pursuit of additional diagnostic testing that may yield few useful data.^20^ In the study by Marton et al,^53^ more than two-thirds of the patients who did well during follow-up had completely normal findings in screening tests, whereas none of the patients who did poorly had normal findings initially. Because organic disease is only rarely found in the patients with normal results from physical examination and laboratory tests, this waiting period is unlikely to result in an adverse out-come.^20^

It is necessary to remember that the technique for assessing body weight should be consistent, so that we are able to compare the measurements. The patient should be weighed with the same amount of clothing on, and at the same time of the day on the same scale each time. Two measurements should be made one after another, and they should agree to within 0.1 kg.

## TREATMENT

### Prevention

A characteristic believed to be typical of older individuals is the significant impairment of the ability to fully recover weight loss that may occur due to acute stressful events such as illness, surgery, or bereavement.^54^ In addition, Moriguti et al.^55^ have shown that healthy older individuals did not significantly regain weight lost due to underfeeding (hypocaloric diet) within the subsequent 6 months, and a decreased perception of hunger was identified as a potential causal factor. Early attention to nutrition and prevention of weight loss during periods of acute stress, particularly during hospitalization, may be extremely important, as efforts directed at re-feeding are often unsuccessful.^36^

### Treatment of causes

The first step in managing patients with weight loss is to identify and treat any specific causative or contributing conditions.

Depression and nonmalignant gastrointestinal diseases are common reversible causes of weight loss.^36^ Treatment of depression often leads to rapid improvements in appetite and intake, and unless contraindicated, tricyclics (which tend to stimulate appetite) may be preferable to serotonin reuptake inhibitors (which tend to suppress appetite) for the treatment of major depression with weight loss. Given the potential difficulties in diagnosing late-life depression, but the excellent potential for reversibility, a trial of therapy should be considered for patients with weight loss and possible depression.

Despite appropriate evaluation, a clear cause for weight loss will often not be found, perhaps because multiple factors, each insufficient to cause weight loss, may coexist and conspire to cause weight loss (e.g. dysgeusia plus visual deficits, and living alone plus lack of knowledge about food preparation). The impact of such factors may also vary greatly, depending on the patient's overall health status, so that even relatively "minor" factors might tax a frail patient beyond their limited ability to compensate, and therefore weight loss ensues (e.g. hyperthyroidism plus anorexia). Efforts should be made to address any factors that appear modifiable, as it may not be necessary to eliminate all contributing factors to help reverse a patient's history of declining weight. Simple measures, such as altering food consistency when oral cavity or dysphagia problems are present (often guided by a speech therapist) may dramatically improve food intake.^56^ Medications should be carefully reviewed, and any that can adversely affect intake should be discontinued (i.e. antihistamines, captopril, carbamazepine, allopurinol, levodopa, digoxin, and theophylline elixir). Assessment of psychosocial factors, including finances, social isolation, and the need for assistance is mandatory. Optimal management often requires multidisciplinary assessment and follow-up (physicians, dentists, dietitians, speech/physical/occupational therapists, social services).

### Nutritional support

A small number of controlled studies have shown positive effects from energy supplementation in undernourished older people. No study has shown energy supplementation as being beneficial in healthy older adults despite numerous advertisements in the lay press suggesting that energy supplements may improve quality of life. A randomized trial of energy supplementation to frail elderly living in the community has shown weight gain and a reduction in falls in the treated group compared with the control group.^57^ Nutritional supplementation in persons with hip fracture has been particularly effective in decreasing morbidity and mortality.^58,59^ A study of oral energy supplementation for older persons in hospital showed a significant decrease in mortality. These controlled trials confirm the epidemiological studies that found that protein-energy malnutrition is associated with increased mortality and prolonged hospital stays and costs.^60,61^

The preferable way to administer food is orally. It is necessary that care be taken to ensure that the patient's ethnic food choices and food preferences are respected. In a single study in which preferred food (ice cream) was allowed ad libitum, protein-energy malnutrition was reversed.^62^

In general, it best to provide energy supplements through the gut whenever possible. In general, enteral tube feeding has a lower complication rate, is associated with more efficient nutrient utilization, is more cost-effective, and is easier to administer than parenteral feeding.^29^ Enteral feeding is indicated in patients with weight loss who can not ingest adequate amounts of food, but have enough gastrointestinal function to allow digestion and absorption of feeding solutions delivered into the gastrointestinal tract through tubes. Peripheral parenteral nutrition offers promise for borderline malnourished patients who are eating poorly, either because of limitations placed on their intake as a result of multiple testing in the hospital or because of anorexia and poor gastrointestinal motility associated with illness.^29^

There are multiple complications associated with re-feeding in older subjects.^63^ A complication that appears to be highly specific to older persons is postprandial hypotension after enteral feeding. Postprandial hypotension has been associated with falls after a meal and with syncope.^64^ The development of postprandial hypotension is secondary to the release of a vasodilator peptide from the gut during feeding.^65^

### Pharmacological Treatment

Despite several medications having been suggested for the treatment of anorexia in older persons, none of these orexigenic drugs has as yet found an established place in the management of protein-energy malnutrition. These drugs, although they can increase appetite and promote weight gain, also have a large range of severe side effects (Table 4), such that they should only be used in selected patients.

In summary, weight loss in elderly people is clearly a prevalent, complex problem. It is associated with an increased risk of morbidity and mortality, and therefore deserves the serious attention of the attending physician. Patients who are identified as being at high risk require immediate intervention, including medical and psychological evaluations. Although such diagnostic uncertainty may be troubling, the prognosis in most cases is surprisingly good. Appropriate social and psychological supports are necessary, and until more is know about the effective treatment of this problem, practical approaches that improve caloric intake should be tried.

**Table 4:** Orexigenic drugs and major side effects

Drug	Side effects
Cyproheptadine	Delirium, sedation, dizziness
Growth hormone	Water retention, arthralgia, carpal tunnel syndrome
Megestrol	Delirium, constipation, water retention
Ornithine oxoglutarate	Hypoglycemia
Tetrahydrocannabinol	Nausea, delirium sedation
Metoclopramide	Parkinsonism, delirium
Cisapride	Abdominal symptoms
Meclobemide	Orthostatic hypotension, delirium
Testosterone (males only)	Elevated hematocrit
Oxandrolone	Liver dysfunction

*After Morley.^29^*
